# Analysis of influencing factors on gas film characteristics for hemispherical dynamic pressure motors

**DOI:** 10.1038/s41598-023-33189-w

**Published:** 2023-04-11

**Authors:** Yaping Zhang, Yanzhong Wang, Fuli Zhang, Wentao Niu, Kai Yang, Boji Lu

**Affiliations:** 1 School of Mechanical Engineering and Automation, Beihang University, Beijing, 10019 China; 2 Beijing Institute of Aerospace Control Devices, Beijing, 100853 China; 3 Laboratory of Science and Technology on Ultra-Precision Aerospace Control Instrument, Beijing, 100853 China; 4 Beijing Spacecraft, Beijing, 100094 China

**Keywords:** Aerospace engineering, Electrical and electronic engineering, Mechanical engineering

## Abstract

The hemispherical dynamic pressure motor (HDPM) has the advantages of high speed, wear resistance and stability, which is widely used in inertial instruments to produce the gyroscopic effect. The ultra-thin gas film between the stator and rotor of the motor provides dynamic pressure lubrication and bearing capacity, whose dynamic characteristics determine the motor performance. However, the influence mechanism of some key factors such as ball center distance on the film characteristics is not clear, which has become the bottleneck restricting the performance improvement of HDPMs. Therefore, in this paper, a series of gas film similarity models were solved under different geometric and working parameters, and the influence law of the ball center distance, rotor displacement and stopping process on the aerodynamic characteristics was obtained, the results show that these primary parameters have significant effects on the pressure distribution, resistance moment and frictional heat of the ultra-thin gas film. This work can not only provide a theoretical basis for the aerodynamic performance optimization of HDPMs, but also serve as a reference for the design of other aerodynamic instruments.

## Introduction

**Figure 1:**
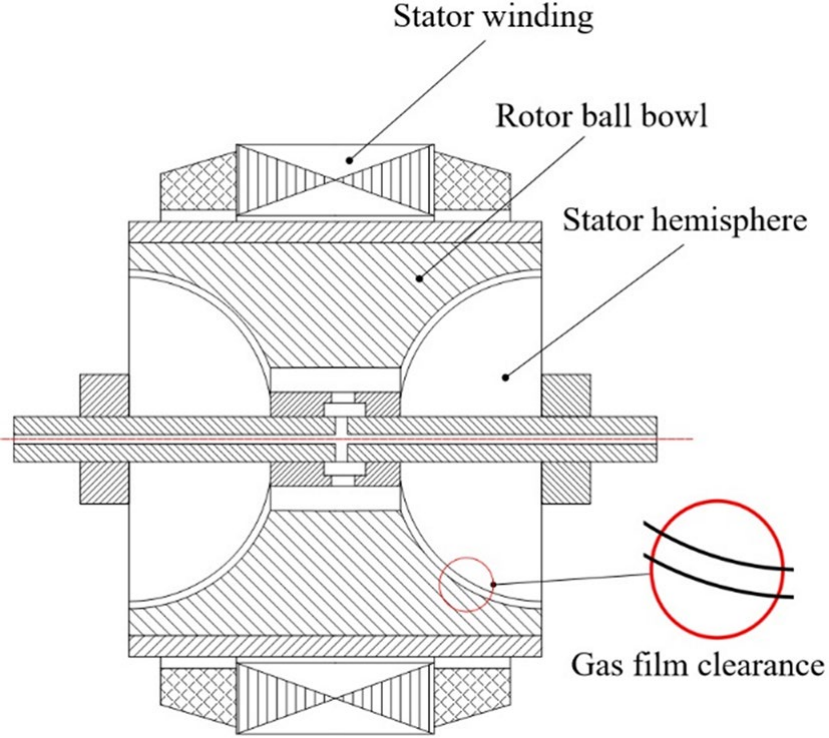
Schematic diagram of hemispherical dynamic pressure motor.

Hemisphere dynamics is an advanced scientific field, which plays an important role in some applications, such as hemispherical resonator gyroscopes (HRGs)^1,2^ and hemisphere dynamic pressure motors (HDPMs)^3,4^. Xu et al. have a lot of involvement in HRGs, they proposed a modeling method for the fully closed-loop system of hemispherical resonator gyroscope and a novel dynamic model of an imperfect hemispherical shell resonator^1,2^. With the advantages of high speed, wear resistance and stability, the HDPM (Fig. 1) is widely used in high-precision gyroscope instruments to produce gyroscopic effect^5,6^. When the HDPM works, the stator winding generates excitation, which drives the rotor ball bowl to rotate at a high speed. The gas is pumped into the clearance through the ball bowl or the etched spiral groove on the hemisphere to form a gas film to realize dynamic pressure lubrication. The clearance between the hemisphere and the ball bowl directly affects the hydrodynamic lubrication performance of the motor, so it is generally necessary to select and match it reasonably^7–9^. For a given size of the hemisphere and ball bowl, the required clearance can be obtained by adjusting the ball center distance between them during assembling. Niu et al. put forward requirements for clearance selection from the perspective of contact characteristics during motor start-up and stop^10^. However, there is no special research report on the characteristics and variation rules of gas film stiffness, resistance torque and friction heat of the gas film under different ball center distances.

Under normal working conditions, the ball bowl will have a slight displacement from the hemispherical rotor for the gravity effect, and the gas film performance of the left and right ends or the upper and lower sides of the same end (Fig. 1) will be different, which may affect the motor temperature and instrument accuracy. When the motor starts or stops, due to the gas dynamic pressure characteristics, the clearance between the hemisphere and the ball bowl will change with the rotating speed. Obtaining the corresponding motor clearance at different speeds is of great significance to analyze the starting and stopping performance of the dynamic pressure motor.

In the past research work^11^, in view of the characteristics of the cross-scale structure of the ultra-thin gas film, we combined the similarity theory with the CFD method, and proposed a method to enlarge the film thickness while maintaining the physical state of the gas film. Based on this theory, a gas film similarity model was established, and the dynamic characteristics of the gas film were simply analyzed under the condition that the motor structure and working parameters were fixed. To provide a practical reference for the design and optimization of HDPMs, this paper has carried out further in-depth research based on the previous work, which revealed the influence of some key factors such as ball center distance and stopping process on the gas film characteristics.

## Calculation model of gas film characteristics

The hemispherical dynamic pressure motor used in this study has a multi-scale structure, and the motor clearance between the hemisphere and the ball bowl is very tiny, which makes the establishment and calculation of the motor model with obvious clearance characteristics very complicated. Therefore, based on the similarity principle^7^, the motor clearance was enlarged by 100 times. On this basis, the CFD method was used to calculate the gas film characteristics of the motor. All calculations involved in this paper adopt the independently developed gas film similarity model of the HDPM, which was described in detail in References 11, so this section only briefly introduces the basic characteristics and primary theory of the model. It should be emphasized that the correctness of the calculation model has been verified by experiments in References 11, so the relevant conclusions obtained in this paper based on the solution of the model can be considered reliable.

### Similarity model

When the actual physical model cannot be established due to the complexity of the structure and boundary conditions, the similarity model is often established based on some principles, so that the characteristic parameters of the actual physical model and the similar model are proportional. Based on the similarity principle^12,13^, the similarity model of the hemispherical dynamic pressure motor was established.

1. Similarity criterion
  1 $$\frac{{\lambda_{h}^{2} \lambda_{p} }}{{\lambda_{\mu } \lambda_{u} }} = 1;\begin{array}{*{20}c} {} \\ \end{array} Re = \frac{uh\rho }{\mu } = \frac{u^{\prime}h^{\prime}\rho ^{\prime}}{{\mu ^{\prime}}};\begin{array}{*{20}c} {} \\ \end{array} Eu = \frac{\Delta p}{{\rho u^{2} }} = \frac{\Delta p^{\prime}}{{\rho ^{\prime}u^{{\prime}{2}} }};\begin{array}{*{20}c} {} \\ \end{array} Ma = \frac{u}{c} = \frac{u^{\prime}}{{c^{\prime}}},$$

where *λ*_*h*_ is the clearance ratio, *λ*_*p*_ is the pressure ratio, *λ*_*μ*_ is the viscosity ratio, *λ*_*u*_ is the velocity ratio, *u* is the velocity, *h* is the gap, *ρ* is the density, *μ* is the viscosity, *c* is the sound velocity, and the superscript ‘'’ represents the corresponding variable in the similar system.

The above equations are the similarity criterion number, Reynolds number, Euler number and Mach number of hydrodynamic lubrication. If two hydrodynamic lubrication systems meet all the requirements of Eq. (1), the two systems are similar.

(2) Similar boundary conditions
  2 $$\lambda_{h} = 100;\lambda_{p} = 1;\lambda_{u} = 1$$
(2) Similarity model

By substituting Eq. (2) into Eq. (1), the similarity ratio of each physical quantity in the two systems was obtained as follows:

3 $$\lambda_{h} = \lambda_{\rho } = \lambda_{t} = 100;\lambda_{\mu } = 10000;\lambda_{p} = 1;\lambda_{u} = 1,$$

where *λ*_*t*_ is the torque ratio.

It can be seen that the similar model enlarged the motor clearance by 100 times, increased the gas density by 100 times, and increased the viscosity by 10,000 times. The aerodynamic parameters such as pressure and velocity obtained by the similar model are the same as those of the original model.

### Governing equations

According to the principle of computational fluid dynamics^14,15^, the governing equations for solving the lubrication gas film of the hemispherical dynamic pressure motor are as follows:

1. Mass conservation equation
  4 $$\frac{\partial \rho }{{\partial t}} + \nabla \cdot \left( {\rho {\mathbf{U}}} \right) = 0,$$

where *t* is the time, ***U*** is the velocity vector of the fluid, and ∇ is Laplace operator.

(2) Momentum conservation equation
  5 $$\frac{{\partial \left( {\rho {\mathbf{U}}} \right)}}{\partial t} + \nabla \cdot \left( {\rho {\mathbf{U}} \otimes {\mathbf{U}}} \right) = - \nabla p + \nabla \cdot {{\varvec{\uptau}}},$$
  where ⊗ is the tensor product operator, and *τ* is the internal shear stress of the fluid.

Because the clearance size of the dynamic pressure motor is in the micron range, and its Reynolds number is small, only laminar flow was considered in the model.

### Geometry parameters and boundary conditions

The parameters of the HDPM were given, including stator hemisphere radius *r*_*b*_, initial clearance *h,* and spiral groove depth *d*. Based on the CFX module of ANSYS/WB, the rotor speed and the axial and radial displacement of the hemisphere are modeled parametrically. The fluid viscosity and density were given in the model, the gas inlet and outlet were set as open boundaries, and the other surfaces were set as nonslip wall surfaces.

## Effect of ball center distance on gas film characteristics

**Figure 2:**
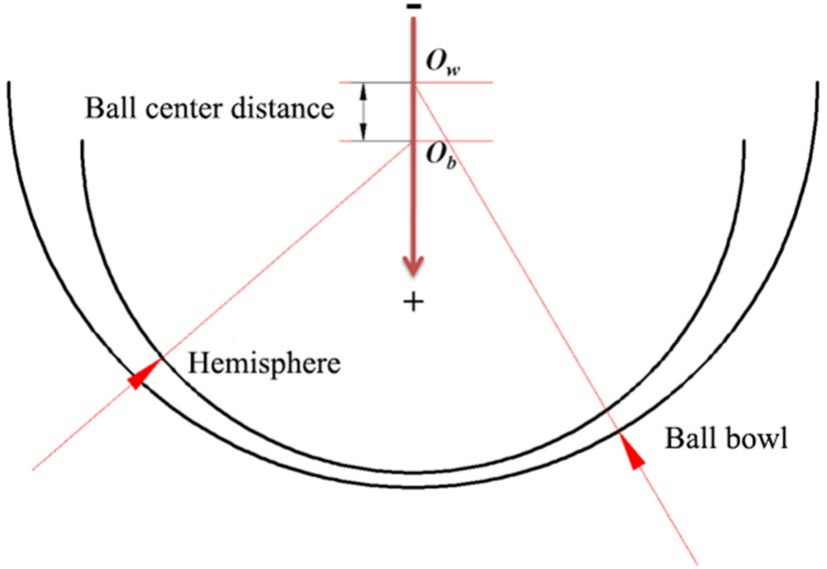
Diagram of ball center distance.

The ball center distance formed by the assembly process of the HDPM is shown in Fig. 2. The ball bowl was fixed, and different ball center distances were obtained by adjusting the position of the hemisphere in the axial direction of the motor. Take the center of the ball bowl *O*_*w*_ as the origin, and if the hemispherical center *O*_*b*_ is offset along the arrow direction, the ball center distance is positive, otherwise, it is negative.

### Calculation results

**Figure 3:**
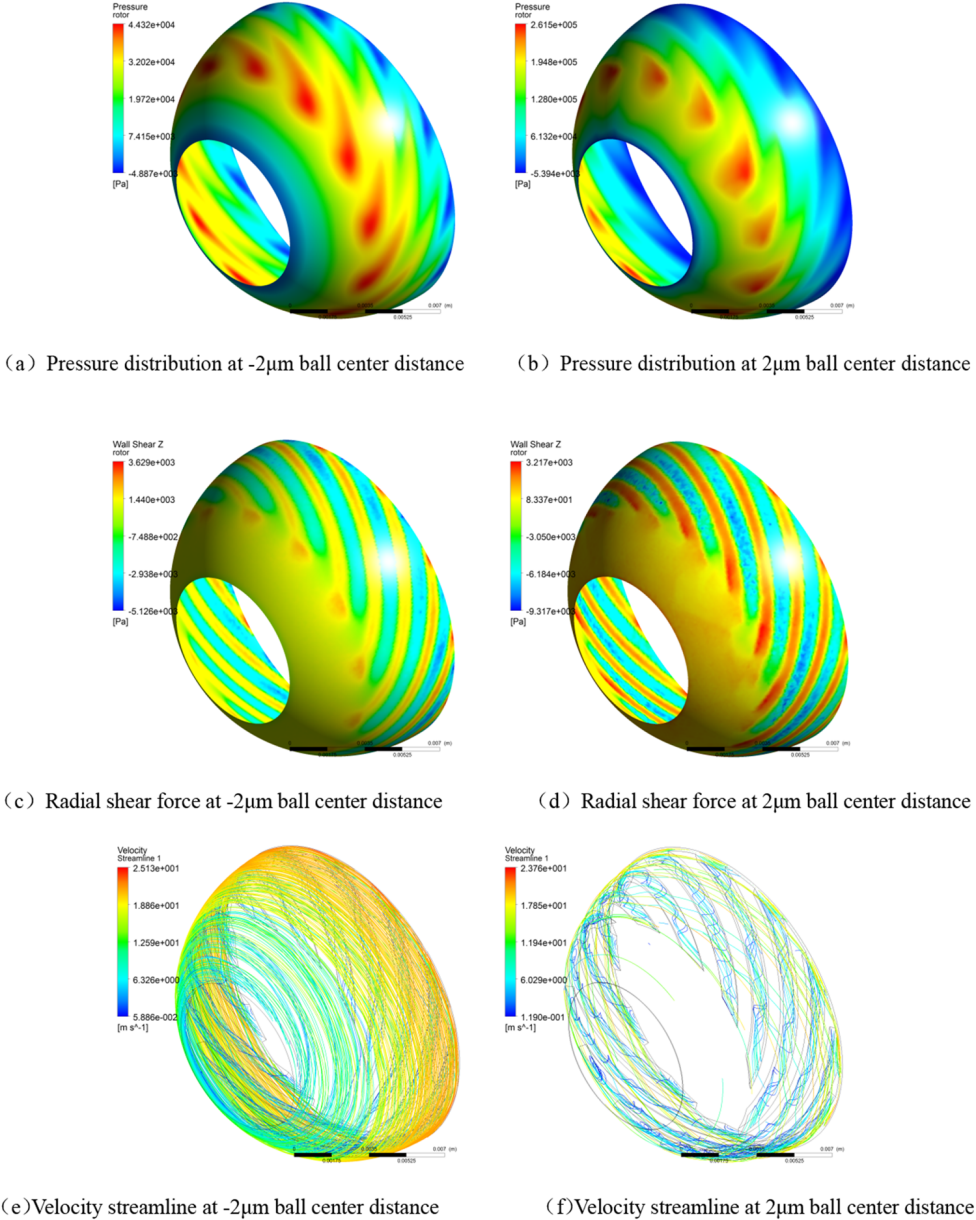
Gas film characteristics at different ball center distances.

The CFX software was used to solve similarity models with different ball center distances, and the graphical results shown in Fig. 3 are obtained.

### Analysis of gas film characteristics

#### Gas film load

**Figure 4:**
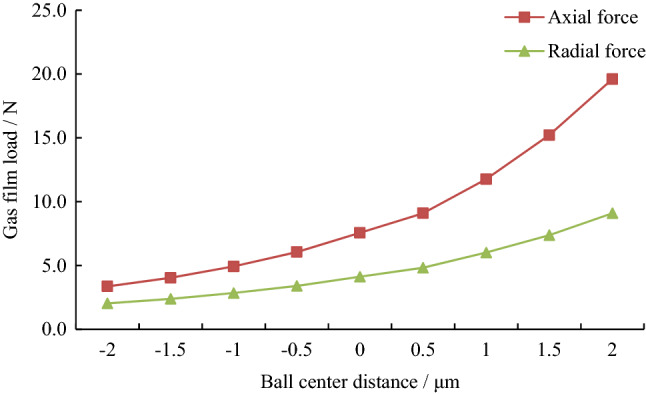
Gas film load under different ball center distances.

The gas film load of hemispheres and ball bowls under different ball center distances was calculated as shown in Fig. 4.

In the figure above, the radial force is the unilateral radial force of a single hemisphere and ball bowl. For two hemispheres and bowls, the radial load is twice the calculated value in the figure. It can be seen from Fig. 4 that with the increase of the ball center distance (the radial clearance increases and the axial clearance decreases), the axial force and radial force increase nonlinearly simultaneously, and the high-pressure zone moves to the small end (Fig. 3a,b).

#### Resistance torque and heat power

**Figure 5:**
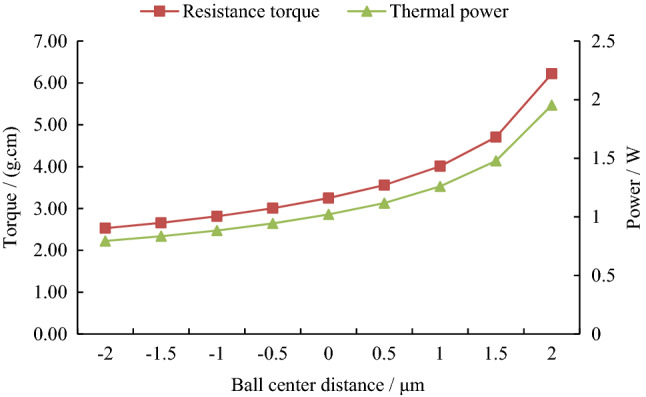
Friction torque and power at different ball center distances.

The resistance torque and thermal power of hemispheres and ball bowls at different ball center distances were calculated as shown in Fig. 5.

It can be seen from Fig. 5 that with the increase of ball center distance (radial clearance increases and axial clearance decreases), the friction resistance torque and the heat generated between the hemisphere and the ball bowl increase exponentially.

#### Gas velocity

**Figure 6:**
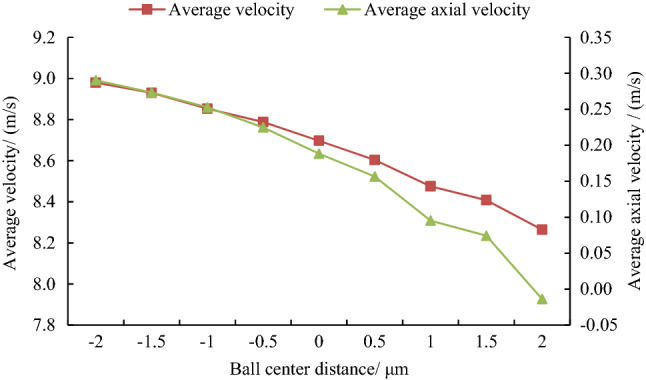
Gas velocity at different ball center distances.

The gas velocity between the hemisphere and the ball bowl under different ball center distances was calculated as shown in Fig. 6.

It can be seen from Fig. 6 that with the increase of the ball center distance (radial clearance increases and axial clearance decreases), the average total velocity and axial velocity of the gas in the gap decrease rapidly. In addition, the maximum axial velocity was about 2.65 m/s.

## Effect of rotor displacement on gas film characteristics

For the hemispherical dynamic pressure motor shown in Fig. 1, the gas film at both ends forms a certain stiffness to balance the rotor weight together during normal operation. Therefore, the rotor will drift along the axial and radial direction under the gravity effect, which will affect the gas friction torque and heat generation in the clearance. In the calculation, the rotor weight of a dynamic pressure motor was taken as *F*.

### Axial displacement of the rotor

When there is acceleration along the axial direction of the motor, the rotor will deviate along the axial direction of the motor. At this time, the axial clearance at one end decreases and the gas film load increases, while the axial clearance at the other end increases and the gas film load decreases. When the difference in axial gas film load is equal to the inertial force of the rotor, the balance state will be reached. The displacement of the rotor under axial force is consistent with the model of different ball center distances in “Effect of ball center distance on gas film characteristics”. Due to the nonlinear change of gas film load with the ball center distance (Fig. 4), the rotor displacement with different ball center distances is different in normal operation. In general, to obtain good clearance matching and friction performance, the ball center distance should be positive when the motor is installed^6^. Therefore, the following two cases were analyzed respectively, i.e., the hemisphere and the ball bowl were concentric, and the ball center distance was 1.5 μm.

#### Concentric hemisphere and bowl

**Figure 7:**
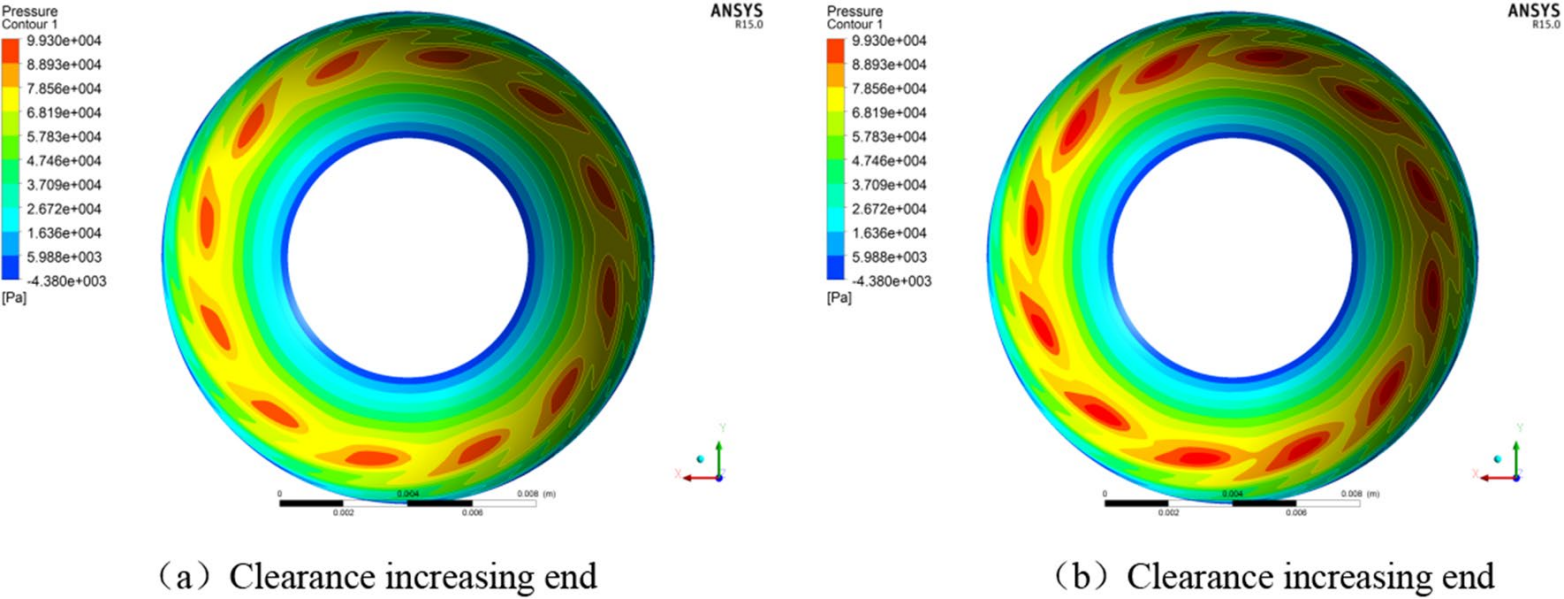
Pressure distribution of gas film under different axial displacements.

When the hemisphere and the ball bowl were concentric, it was calculated by using the model in “Effect of ball center distance on gas film characteristics” that the axial displacement of the rotor under gravity effect was 0.11 μm (the clearance at one end decreases and at the other end increases), and the axial resultant force at both ends was *F*. At this time, the gas film characteristics at both ends are shown in Fig. 7 and Tab. 1.

**Table 1:** Gas film characteristics at both ends of the rotor with 0.11 μm axial displacement.

	Resistance torque *T* (g cm)	Friction heat power *P* (W)
One end: clearance decreases by 0.11 μm	3.31	1.0396
The other end: clearance increases by 0.11 μm	3.19	1.0024
Total	6.50	2.0420

It can be seen that if the hemisphere and the ball bowl are concentric, the supporting force provided by the gas film at both ends can offset the rotor weight when the rotor is axially deviated by 0.11 μm, that is, the axial deviation of the rotor under the action of gravity is about 0.11 μm. In this state, the friction thermal power of the gas film at the small clearance end is larger than that at the big clearance end by 37.2 mW, which may cause the difference of temperature and thermal deformation at both ends, and affect the instrument accuracy (mass center changes along the motor axis) or motor reliability.

#### Ball center distance of 1.5 μm

When the ball center distance was 1.5 μm, the axial displacement of the rotor under gravity was 0.02 μm. The gas film characteristics at both ends are shown in Table 2.

**Table. 2:** Gas film characteristics at both ends of the rotor with an axial offset of 0.02 μm.

	Resistance torque *T* (g cm)	Friction heat power *P* (W)
One end: clearance decreases by 0.02 μm	4.74	1.4898
The other end: clearance increases by 0.02 μm	4.67	1.4663
Total	9.41	2.9561

It can be seen from Table 2 that if the ball center distance is 1.5 μm, the supporting force provided by the gas film at both ends can offset the weight of the rotor after the axial deviation of the rotor is 0.02 μm, that is, the axial deviation of the rotor under the action of gravity is about 0.02 μm during normal operation. In this state, the friction thermal power of the gas film at the small clearance end is larger than that at the big clearance end by 23.5 mw, which may also cause the difference in temperature and thermal deformation at both ends.

Compared with the calculation results of the concentric hemisphere and the ball bowl and the ball center distance of 1.5 μm, it can be concluded that the larger the ball center distance (the closer the hemisphere is to the ball bowl), the smaller the axial displacement required to balance the rotor and the smaller the thermal power difference between the two ends.

### Radial displacement of rotor

When the gravity is along the radial direction of the motor, the rotor will move along the radial direction of the motor, supported by hemispheres and bowls at both ends. For hemispheres and bowls at either end, the radial clearance of half of them decreases and the gas film load increases, while the radial clearance of the other half increases and the gas film load decreases. When the difference of radial load on gas film at both ends is equal to the gravity of the rotor, the balance state is reached.

**Figure 8:**
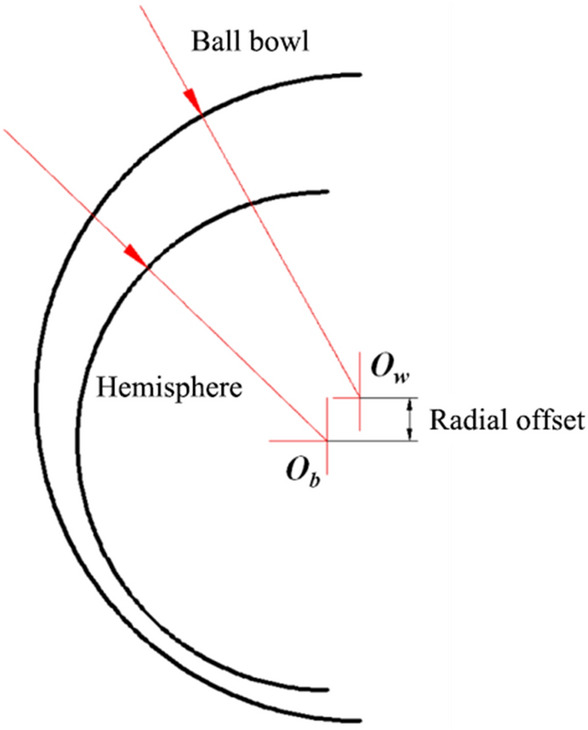
Radial offset of motor.

The three-dimensional model of the motor with the rotor radial offset as shown in Fig. 8 was established, and two cases of the concentric hemisphere and ball bowl and 1.5 μm ball center distance were analyzed respectively.

Similarly, assuming that the rotor weight of the dynamic pressure motor is *F*, the support force to be provided at the single end is *F*/2.

#### Concentric hemisphere and bowl

**Figure 9:**
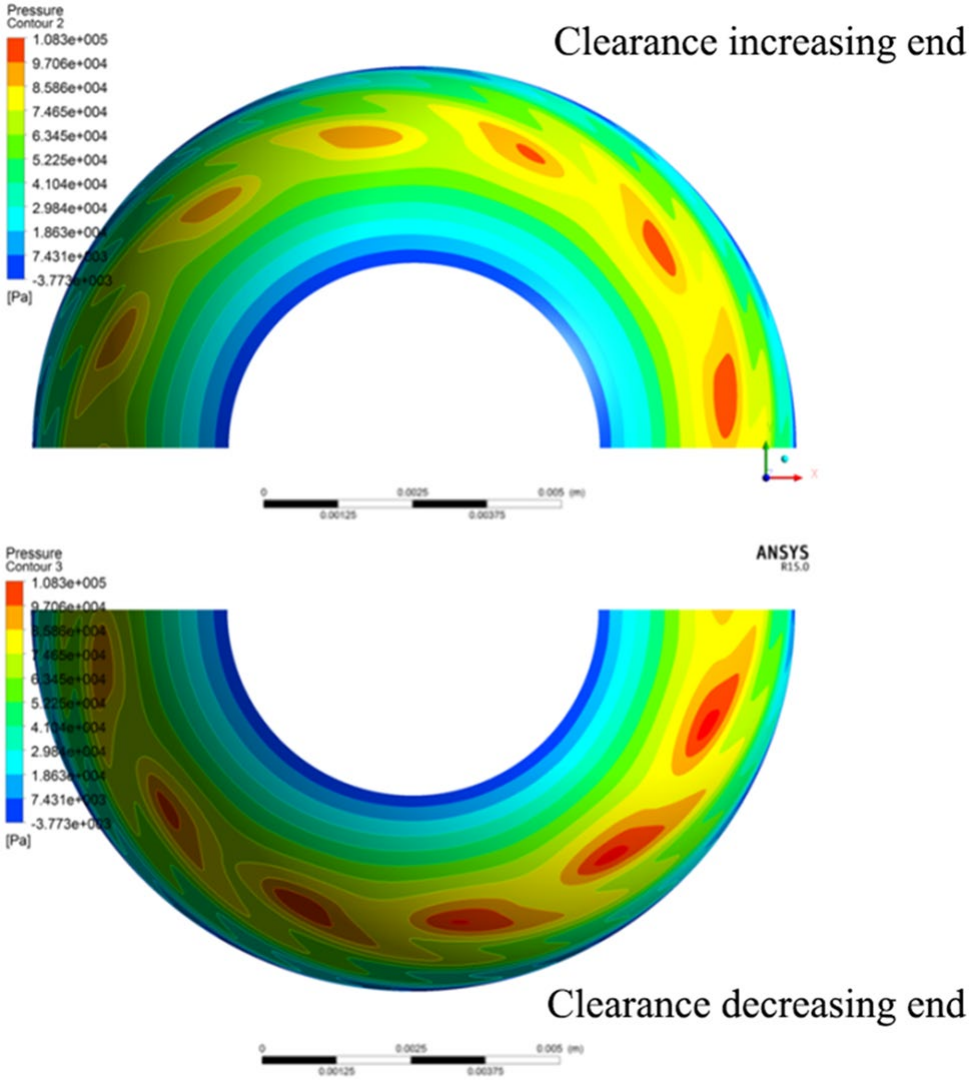
Pressure distribution of gas film under different radial offsets.

When the hemisphere and the ball bowl were concentric, the gas film characteristics of the two half sides were calculated. The rotor radial displacement under the gravity effect was 0.08 μm (the half clearance decreases and the other half clearance increases), and the corresponding gas film characteristics are shown in Fig. 9 and Table 3.

**Table 3:** Gas film characteristics of two half sides at the same end of the rotor with 0.08 μm radial offset.

	Resistance torque *T* (g.cm)	Friction heat power *P* (W)
One half side: clearance decreases by 0.08 μm	1.65	0.5184
The other half side: clearance increases by 0.08 μm	1.60	0.5027
Total	3.25	1.0211

It can be seen from Fig. 9 and Table 3 that if the hemisphere and the ball bowl are concentric, the supporting force provided by the single-end gas film is half of the weight of the rotor after the radial offset of the rotor is 0.08 μm under the action of gravity. In this state, the friction heat power of one side of the small clearance is 15.7 mW larger than that of the other side, which may cause the temperature and thermal deformation difference of the same hemisphere and the ball bowl in the radial direction, resulting in the mass center changing along the radial direction of the motor.

#### Ball center distance of 1.5 μm

When the ball center distance was 1.5 μm, the radial displacement of the rotor under gravity was 0.02 μm, and the gas film characteristics of the two half sides are shown in Table 4.

**Table 4:** Gas film characteristics of two half sides at the same end of the rotor with 0.02 μm radial offset.

	Resistance torque *T* (g cm)	Friction heat power *P* (W)
One half side: clearance decreases by 0.02 μm	2.37	0.7446
The other half side: clearance increases by 0.02 μm	2.34	0.7351
Total	4.71	1.4797

It can be seen from the analysis in the above table that if the ball center distance is 1.5 μm, the supporting force provided by the gas film at both sides is half of the weight of the rotor after the radial offset of the rotor is 0.02 μm. Under this condition, the friction thermal power of the gas film of the small clearance side is 9.5 mW larger than that of the other side, which may also cause the temperature and thermal deformation difference of the same hemisphere and the ball bowl.

By comparing the calculation results of the concentric hemisphere and the ball bowl and the ball center distance of 1.5 μm, a similar conclusion as the axial force analysis in the “Axial displacement of the rotor” can be obtained: the larger the ball center distance, the smaller the axial offset required to balance the rotor, and the smaller the thermal power difference between the two half sides of the same end.

## Effect of motor stopping process on gas film characteristics

During the stop of the dynamic pressure motor, the gas film clearance will decrease with the decrease of the rotating speed to balance the weight of the rotor until the stator and rotor contact at the minimum clearance. Under the conditions of the concentric hemisphere and ball bowl and 1.5 μm ball center distance, the changes of the clearance and resistance torque, and the near contact speed of the motor in the process of stalling are discussed.

### Clearance and resistance torque

**Figure 10:**
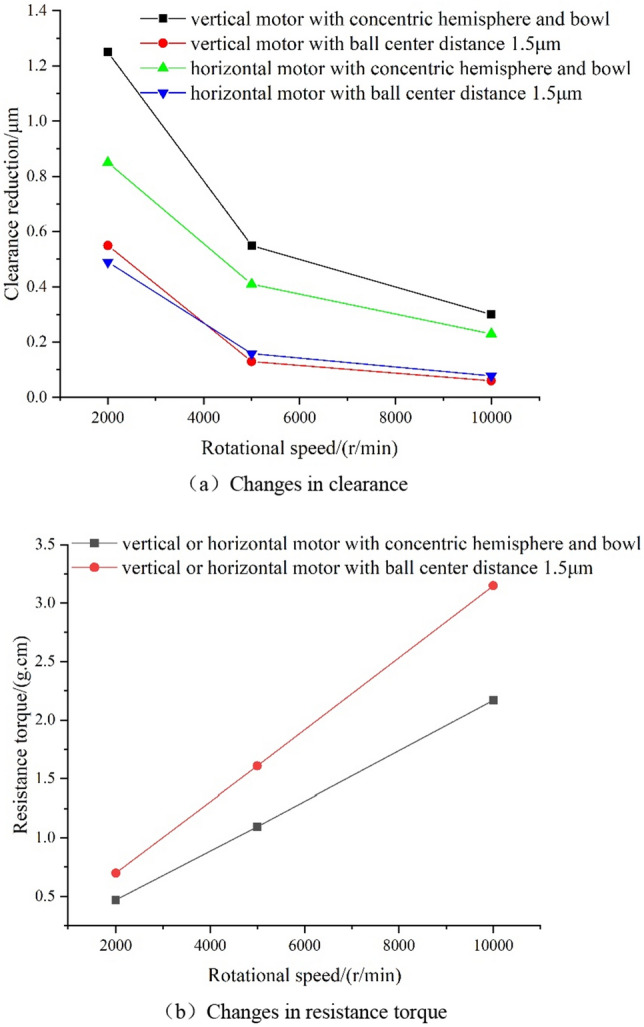
Changes of clearance and resistance torque during motor stopping.

When the motor stops running, the clearance at one end (axial) or one side (radial) gradually decreases, while that at the other end (axial) or one side (radial) increases gradually. The corresponding clearance and resistance torque were calculated iteratively when the motor stops running vertically and horizontally, as shown in Fig. 10.

As can be seen from the above figure:

1. The clearance decreases with the decrease in rotating speed. When the speed of the motor in a vertical state drops to 2000 r/min, the clearance decreases by 0.55 μm (ball center distance 1.5 μm) and 1.25 μm (concentric) respectively. When the motor speed drops to 2000 r/min in a horizontal state, the clearance decreases by 0.49 μm (ball center distance 1.5 μm) and 0.85 μm (concentric) respectively.
2. The resistance torque is directly proportional to the speed of the motor, and has nothing to do with the vertical or horizontal state of the motor; the larger the ball center distance is, the greater the resistance torque is, and the faster it changes with the speed.

### Critical contact speed

**Figure 11:**
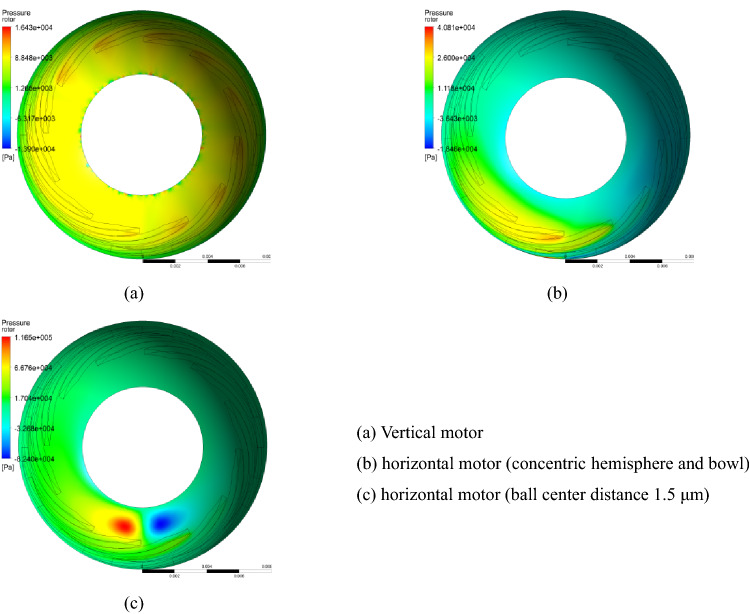
Pressure distribution of gas film under critical contact state.

According to the method in Ref.^6^, the minimum axial and radial clearances are 2.3 μm and 2.0 μm when the hemisphere and the ball bowl are concentric, and 0.8 μm and 1.3 μm when the ball center distance is 1.5 μm. The motor speed with the minimum clearance of 0.01 μm was considered as the critical contact speed, on this basis, the critical contact speed and resistance torque under various conditions could be calculated, as shown in Table 5 and Fig. 11.

**Table 5:** Critical contact speed and resistance torque of motor under different conditions.

Motor states	Stopping in the vertical state		Stopping in the horizontal state	
Analysis items	Critical contact speed (r/min)	Resistance torque (g cm)	Critical contact speed (r/min)	Resistance torque (g cm)
Concentric hemisphere and bowl	780	0.35	1275	0.77
Ball center distance 1.5 μm	1170	0.57	1300	0.97

Under the condition of the adopted structural parameters, the critical contact speed of the horizontal motor is larger than that of the vertical motor, but with the increase of ball center distance, the critical contact speed of the vertical motor increases significantly, and the change of horizontal motor is small, and the difference between them decreases. In addition, the gas resistance torque during the vertical motor stopping is smaller than that of the horizontal motor stopping. In addition, the greater the ball center distance is, the greater the gas resistance torque.

## Conclusions

Based on the independently developed gas film similarity model of the HDPM, this paper analyzed the gas film characteristics under different structure and operating parameters, thus the influence rules of ball center distance, rotor displacement, stopping process, and other key factors on gas film characteristics are obtained, which could provide a practical reference for the design and optimization of HDPMs. Through the study of this paper, the following beneficial conclusions can be obtained:

1. With the increase of the ball center distance (i.e., the radial clearance increases and the axial clearance decreases), the gas film load, resistance torque and thermal power of the motor increase exponentially, the high-pressure zone moves to the small end, and the average gas velocity decreases.
2. The larger the ball center distance is, the smaller the axial and radial offsets are, and the smaller the thermal power difference between the two ends is.
3. The gas resistance torque in the clearance is directly proportional to the rotational speed, and has nothing to do with the vertical or horizontal state of the motor; the larger the ball center distance is, the greater the resistance torque is, and the faster it changes with the speed.
4. The critical contact speed of the horizontal motor is larger than that of the vertical motor, but with the increase of ball center distance, the critical contact speed of the vertical motor increases significantly and the change of critical contact speed of the horizontal motor is small.
5. During the stopping process, the gas resistance torque of the vertical motor is smaller than that of the horizontal motor, and the larger the ball center distance is, the greater the gas resistance torque is.

## Data Availability

The datasets used and/or analyzed during the current study available from the corresponding author on reasonable request.
